# The lip split: a retrospective outcomes study of central and lateral lip split access for head and neck reconstructive surgery

**DOI:** 10.1007/s10006-025-01355-1

**Published:** 2025-03-06

**Authors:** Peter Gearing, Maxim Devine, Siyuan Pang, Felix Sim, Anand Ramakrishnan

**Affiliations:** 1 Department of Plastic & Reconstructive Surgery, The Royal Melbourne Hospital, 300 Grattan St., Parkville, VIC 3052 Australia; 2 Department of Oral & Maxillofacial Surgery, The Royal Melbourne Hospital, Parkville, VIC Australia; 3 Department of Surgery, The Royal Melbourne Hospital, The University of Melbourne, Melbourne, Australia

**Keywords:** Head and neck, Surgical access, Lip split mandibulotomy, Malignancy

## Abstract

**Purpose:**

Surgical resection of oral cancers requires meticulous planning to achieve clear margins and minimize potential morbidity. This study aimed to compare postoperative surgical and functional outcomes following central and lateral lip-split approaches used for resection and reconstruction of oral tumours.

**Methods:**

A retrospective review of 79 cases involving lip-split procedures for head and neck cancers was conducted. Data were collected from a prospectively recorded database (December 2015 to December 2022). Statistical analyses compared patient demographics, intraoperative characteristics, and postoperative outcomes between central and lateral lip-split cohorts.

**Results:**

Lateral lip splits were associated with higher rates of postoperative complications (*p* = 0.008), including return to theatre (*p* = 0.015), and functional issues including asymmetric smile (*p* = 0.009). No significant differences were observed in readmission rates, length of stay, or time to oral diet commencement (*p* > 0.05).

**Conclusions:**

Lip-split procedures remain valuable for resection and reconstruction of oral and oropharyngeal tumours. Lateral lip splits are associated with poorer outcomes when compared to central approaches. Appropriate selection of lip splitting approaches should consider tumour location, resection margins, patient comorbidities and preferences, and surgical preferences.

## Background

Surgical resection of oral cancers is the mainstay of treatment, with clear margins strongly associated with improved prognosis [1]. Surgical access for oral cavity cancer resection requires balancing the advantages of extensive incisions, which improve visibility and facilitate resection and reconstruction, with the associated functional and cosmetic morbidities. The limited anatomical environment adds challenges, particularly for accessing the posterior structures of the oral cavity and oropharynx, as well as the vasculature required for anastomosis and inset of free vascularized flaps [2]. Purely transoral approaches are effective for many anterior resections and reconstructions [3]. Where transoral approaches are not technically viable or appropriate for obtaining clear pathological margins or inset of free flaps, various alternative approaches have been proposed, including the lip-split mandibulotomy approach (LSMA) first described in 1836 by Roux.

**Fig. 1:**
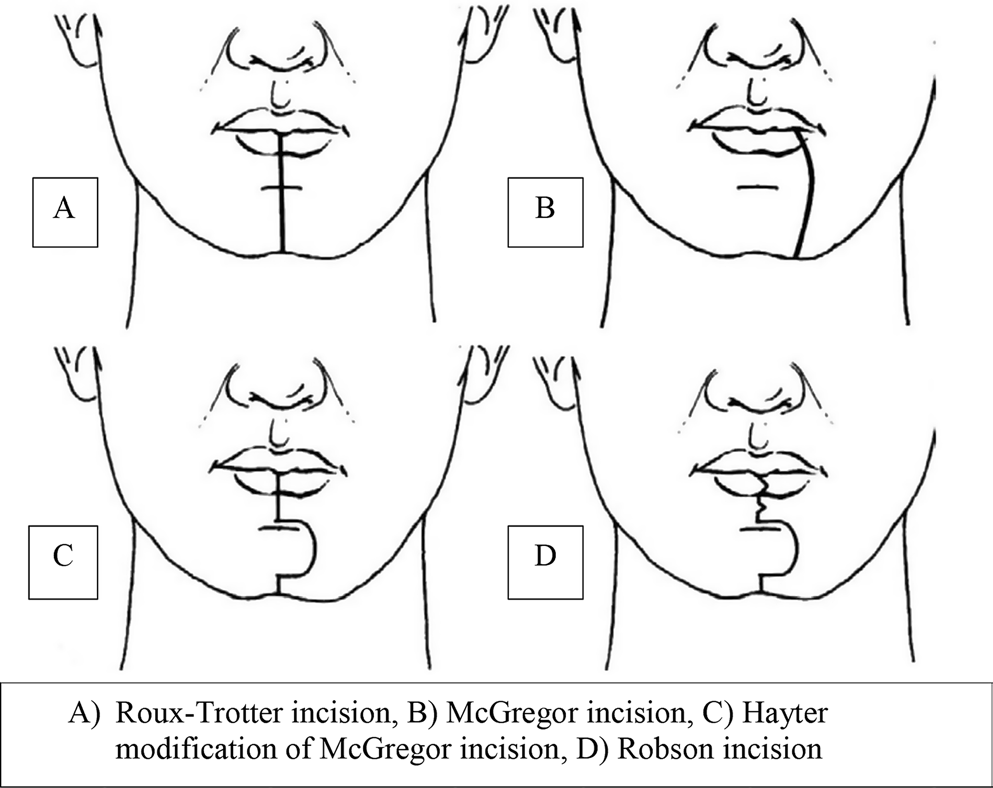
Schematic representations of lip split incisions (adapted from source: Bhatt et al [4])

Popularized in the 1970s, the optimal approach for splitting the lip is debated in literature. Various incisions have been proposed including the Roux-Trotter incision, McGregor incision, Robson incision and the zigzag incision (Fig. 1) [4, 5]. Hayter et al. describe a modification of the McGregor incision with a chevron incision in the vermillion border [4], and Mehanna et al. later described an additional zigzag incision to the chin [5]. These approaches aimed to reduce the cosmetic and functional morbidity associated with conventional midline lip splits, reducing scar formation, and preserving lip and chin mobility [5, 6]. The Robson lip split incision is a lateral modification, to attempt to reduce scar contracture and its associated complications but inevitably compromises mental and facial nerve branches traversing the incision plane [4, 7, 8]. The Weber-Ferguson approach is a comparable approach used to split the upper lip for maxillectomy resection and reconstruction, and may be complicated by significant facial scarring, ectropion, and epiphora.

Despite the variety in techniques, limited literature compares the outcomes of midline and lateral lip splits. This study aims to address this gap by analyzing outcomes from a prospectively collected database of head and neck reconstructive surgeries at a tertiary hospital [2, 9–11].

## Methodology

### Study design and population

This retrospective comparative cohort study utilised data prospectively collected from the RMH head and neck surgical database (December 2015–December 2022). Cases were included if lip-split access was documented for tumour resection and excluded for trauma-related reconstruction. This study was approved by the RMH Human Research Ethics Committee (HREC QA2024011), in accordance with the ethical standards of the 1964 Helsinki Declaration and its later amendments. The RMH head and neck surgical database was screened by three researchers (PG, MD, and CP) for cases occurring between 16th December 2015, and 1st December 2022. Of 488 head and neck reconstructive operations performed in this period, 79 cases were identified as having included a lip split procedure.

### Variables

Predictor Variable: Lip split type (central vs. lateral).

Primary Outcome: Postoperative surgical complications within 30 days, including wound dehiscence, infections, hematoma, and flap failure.

Secondary Outcomes: operating duration, length of stay, surgical site infections, wound dehiscence, flap failure, days to oral intake, oral intake prior to discharge, returns to theatre, readmissions, lip notching, altered smile, altered speech, and oral incompetence (as evaluated in postoperative clinical reviews).

Demographic Variables: age, sex, ASA classification, smoking history, diabetes status, and percutaneous endoscopic gastrostomy (PEG) tube insertion.

Pathological Data: Tumour type, TNM stage, and clinical N-stage were extracted.

Operative Data: Variables included type of resection (marginal, segmental, or mandibulotomy), flap type (soft tissue vs. bony), skin excision, operative duration, and preoperative interventions such as PEG insertion.

A small number of missing datapoints for time to onset of dietary intake were then identified retrospectively from the *Epic* Electronic Medical Record (EMR). Further retrospective data collection included the reasons for return to theatre (RTT) and for readmission.

Inclusion criteria: head and neck reconstruction for tumor resection, lip split access documented.

Exclusion criteria: traumatic reconstruction.

### Statistical analysis

Demographic and clinical variables were tabulated and stratified by the predictor variable (lip split type). Continual variables were reported using means and standard deviations and analyzed with the student’s t-test and Mann–Whitney U test. Categorical variables were analyzed using the Chi^2^ test. Logistic regression models were used to explore associations, controlling for confounders. Statistical analyses were performed using SPSS 27, with p values < 0.05 considered statistically significant.

## Results

### Demographics

Of 488 head and neck reconstructive operations performed between 16th December 2015, and 1st December 2022, 79 cases were identified as including a lip split procedure (16.2% of total cases). Of the 79 lip split procedures, 35 were lateral lip splits (44.3% of lip split cases) and 44 were central lip splits (55.7%) (Table 1). Most patients requiring lip split were male (68.4%) with mean age 62 years and were ASA class 3 (58.9%), with an active or past smoking history (55.7%), and non-diabetic (87.3%). A pre-operative percutaneous endoscopic gastrostomy (PEG) tube was indicated in 15.2% of cases. Operations were predominantly undertaken for malignancy (96.2%), with squamous cell carcinoma excised in 83.5% of cases. Tumours were typically of high T-classification, with 45 cases of T4 cancer (59.2%). Approximately half of cases were clinically node negative at time of resection (52.6%). The mean operative duration was 646 ± 112 min. A mandibulotomy was performed in 16 cases (20.3%) with 10 being central mandibulotomies (12.7%) and 6 lateral mandibulotomies (7.6%). A marginal mandibulectomy was performed in 14 cases (17.7%) and segmental mandibulectomies were performed in 38 cases (48.1%) of which 14 cases were segmental mandibulectomy with disarticulation of the temporomandibular joint (17.7%). The resection margins required skin excision in 4 cases (5.1%). Half of cases were reconstructed with soft tissue free flaps (50.6%) and the rest with bony free flaps (49.4%).

When demographics were compared between patients that underwent lateral and central lip splits the cohorts were largely comparable. There was a significantly higher mean ASA classification in the lateral lip split cohort (*p* = 0.025). There was no statistically significant difference in sex, smoking history, diabetes status, or requirement for pre-operative PEG insertion (*p* > 0.05). The tumour type, clinical T-stage (cT), and clinical N-stage (cN) were also comparable between groups (*p* > 0.05). If mandibulotomy was performed, the site of mandibulotomy was related to the site of lip split (*p* = 0.002). There were significantly more segmental mandibulectomies in the lateral lip split cohort (*p* = 0.002). There was no difference in the rate of marginal mandibulectomy (*p* = 0.904) or skin excision (*p* = 0.205). There was no difference in the type of flap used (soft tissue or bony free flap; *p* = 0.918) nor the mean operative duration (*p* = 0.981).

**Table 1:** Demographic & operative characteristics of included patients according to type of lip split

Characteristic (ordinal variables)		Total *n* = 79 (%)	Lateral lip split *n* = 35 (%)	Central lip split *n* = 44 (%)	*P* value (Chi^2^)
Sex	Male	54 (68.4)	25 (71.5)	29 (65.9)	*p* = 0.600
	Female	25 (31.6)	10 (28.6)	15 (34.1)	
ASA status	1	3 (4.1)	0 (0)	3 (7.0)	*p* = 0.025
	2	27 (37)	7 (23.3)	20 (46.5)	
	3	43 (58.9)	23 (76.7)	20 (46.5)	
Smoking history	Yes	44 (55.7)	20 (57.1)	24 (54.5)	*p* = 0.817
	No	35 (44.3)	15 (42.9)	20 (45.5)	
Diabetes	Yes	10 (12.7)	5 (11.4)	5 (11.4)	*p* = 0.698
	No	69 (87.3)	30 (85.7)	39 (88.6)	
Pre-operative PEG insertion	Yes	12 (15.2)	4 (11.4)	8 (18.2)	*p* = 0.406
	No	67 (84.8)	31 (88.6)	36 (81.8)	
Tumour type	SCC	66 (83.5)	29 (82.9)	37 (84.1)	*p* = 0.903
	Adenocarcinoma	2 (2.5)	1 (2.9)	1 (2.3)	
	Salivary gland carcinoma	5 (6.3)	3 (8.6)	2 (4.5)	
	Sarcoma	2 (2.5)	1 (2.9)	1 (2.3)	
	Ameloblastoma	2 (2.5)	1 (2.9)	1 (2.3)	
	Ameloblastic carcinoma	1 (1.3)	0 (0.0)	1 (2.3)	
	Odontogenic myxoma	1 (1.3)	0 (0.0)	1 (2.3)	
Malignant tumour	Yes	76 (96.2)	34 (97.1)	42 (95.5)	*p* = 0.697
	No	3 (3.8)	1 (2.9)	2 (4.5)	
cT-stage	1	3 (3.9)	2 (5.9)	1 (2.4)	*p* = 0.737
	2	17 (22.4)	6 (17.6)	11 (26.2)	
	3	11 (14.5)	5 (14.7)	6 (14.3)	
	4	45 (59.2)	21 (61.8)	24 (57.1)	
cN-stage	Negative (N0)	40 (52.6)	15 (44.1)	25 (59.5)	*p* = 0.181
	Positive (N1+)	36 (47.4)	19 (55.9)	17 (40.5)	
Mandibulotomy	No	63 (79.7)	29 (82.9)	34 (77.3)	*p* = 0.014
	Central	10 (12.7)	1 (2.9)	9 (20.5)	
	Lateral	6 (7.6)	5 (14.3)	1 (2.3)	
Marginal mandibulectomy	Yes	14 (17.7)	6 (17.1)	8 (18.2)	*p* = 0.904
	No	65 (82.3)	29 (82.9)	36 (81.8)	
Segmental mandibulectomy	Segmental + disarticulation	14 (17.7)	7 (20.0)	7 (15.9)	*p* = 0.002
	Segmental	24 (30.4)	17 (48.6)	7 (15.9)	
	No	41 (51.9)	11 (31.4)	30 (68.2)	
Skin excision	Yes	4 (5.1)	3 (8.6)	1 (2.3)	*p* = 0.205
	No	75 (94.9)	32 (91.4)	43 (97.7)	
Flap type	Soft tissue only	40 (50.6)	17 (48.6)	23 (52.3)	*p* = 0.918
	Bony	39 (49.4)	18 (51.4)	21 (47.7)	
Characteristic (continuous variables)		**Mean ± SD**	**Mean ± SD**	**Mean ± SD**	**P value (t-test)**
Age (years)		62.0 ± 13.4	65.0 ± 12.8	59.6 ± 13.5	*p* = 0.076
Operative duration (mins)		646 ± 112	646 ± 118	650 ± 108	*p* = 0.981

### Lip splits over time

**Fig. 2:**
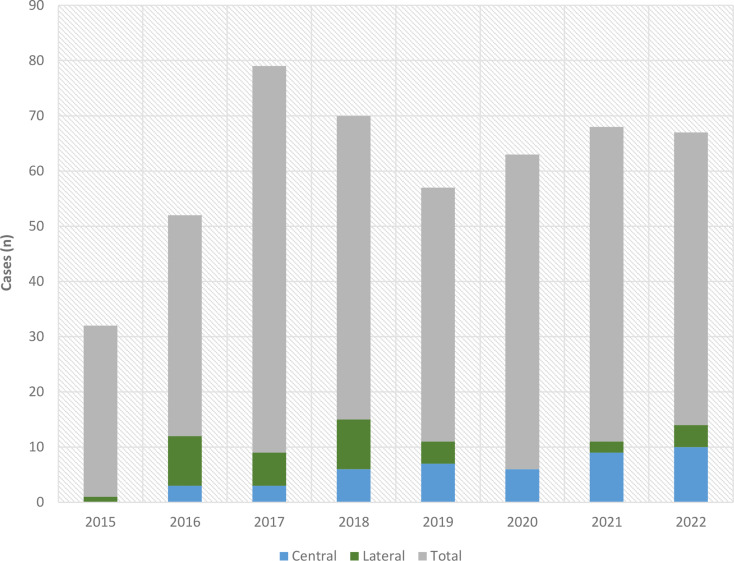
Lip split procedures as a proportion of total procedures per year

The number of total reconstructive cases in the reconstructive head and neck database was relatively consistent over the years studied (mid-2015 to 2022); there was a mean of 61 cases per year with a range of 32 to 79 cases. Figure 2 demonstrates the trend in case load over time, with the subset of central and lateral lip splits highlighted. From 2015 to 2018, predominantly lateral lip splits were performed (67.6%), while from 2019 to 2022 there were predominantly central lip splits (76.2%).

### Outcomes & complications

Postoperative outcomes were compared between patients that underwent lateral and central lip splits (Table 2). Lateral lip splits were significantly associated with complications within 30 days postoperatively (*p* = 0.008) and trended towards a higher rate of postoperative collections (including hematoma, seroma, and abscess) (40% vs. 25%; *p* = 0.154). Lateral lip split operations had a higher rate of return to theatre within 30 days postoperatively (51.4% vs. 25%; *p* = 0.015). There was a trend to higher mean number of returns to theatre with lateral lip splits (0.86 vs. 0.41; *p* = 0.100). Most returns to theatre were for recipient site complications including collections. There was no significant difference in readmission rates within 30 days (*p* = 0.802), with most readmissions again secondary to recipient site complications. There was no significant difference in mean length of stay (21.8 vs. 21.6 days; *p* = 0.921), mean days to oral diet commencement (15.2 vs. 13.1 days; *p* = 0.314), nor the rate of oral incontinence documented in follow-up (*p* = 0.522). Speech intelligibility was also comparable between groups (*p* = 0.522). Lip and smile outcomes were significantly poorer in the lateral lip split cohort. Lip notching was significantly more common after lateral lip splits (*p* = 0.012), as was asymmetric smiling (*p* = 0.009).

**Table 2:** Outcomes according to type of lip split

Outcomes (ordinal variables)		Total *n* (%) *n* = 79	Lateral lip split (%) *n* = 35	Central lip split (%) *n* = 44	*P* value (Chi^2^)
Any complication (< 30 days postop)	Yes	71 (89.9)	35 (100.0)	36 (81.8)	*p* = 0.008
	No	8 (10.1)	0 (0.0)	8 (18.2)	
Post-operative collection (< 30 days)	Yes	25 (31.6)	14 (40.0)	11 (25.0)	*p* = 0.154
	No	54 (68.4)	21 (60.0)	33 (75.0)	
Superficial infection (< 30 days)	Yes	12 (15.2)	4 (11.4)	8 (18.2)	*p* = 0.406
	No	67 (84.8)	31 (88.6)	36 (81.8)	
Flap failure (< 30 days)	Complete	1 (1.3)	1 (2.9)	0 (0.0)	*p* = 0.379
	Partial	13 (16.5)	7 (20.0)	6 (13.6)	
	No	65 (82.3)	27 (77.1)	38 (86.4)	
Oral intake prior to discharge	Yes	71 (89.9)	31 (88.6)	40 (90.9)	*p* = 0.732
	No	8 (10.1)	4 (11.4)	4 (9.1)	
Return to theatre (< 30 days)	Yes	29 (36.7)	18 (51.4)	11 (25.0)	*p* = 0.015
	No	50 (63.3)	17 (48.6)	33 (75.0)	
Return to theatre reason	Flap failure	2 (2.5)	1 (2.9)	1 (2.3)	*p* = 0.086
	Other recipient site complication	25 (31.6)	16 (45.7)	9 (20.5)	
	Donor complication	1 (1.3)	1 (2.9)	0 (0.0)	
	Positive margins	1 (1.3)	0 (0.0)	1 (2.3)	
Readmission (< 30 days)	Yes	26 (32.9)	11 (31.4)	15 (34.1)	*p* = 0.802
	No	53 (67.1)	24 (68.6)	29 (65.9)	
Readmission reason	Flap failure	1 (1.3)	1 (2.9)	0 (0.0)	*p* = 0.313
	Other recipient site complication	11 (14.1)	7 (20.0)	4 (9.3)	
	Donor complication	6 (7.7)	1 (2.9)	5 (11.6)	
	Sepsis	1 (1.3)	0 (0.0)	1 (2.3)	
	Pain	1 (1.3)	0 (0.0)	1 (2.3)	
	Malnutrition	4 (5.1)	1 (2.9)	3 (7.0)	
	Trauma	1 (1.3)	1 (2.9)	0 (0.0)	
Oral incontinence	Yes	26 (32.9)	11 (31.4)	15 (34.1)	*p* = 0.522
	No	52 (65.8)	23 (65.7)	29 (65.9)	
Speech	Dysarthria	29 (36.7)	13 (37.1)	16 (36.4)	*p* = 0.522
	Intelligible	49 (62.0)	21 (60.0)	28 (63.6)	
Smile	Asymmetrical	10 (12.7)	4 (11.4)	6 (13.6)	*p* = 0.009
	Normal	22 (27.8)	4 (11.4)	18 (40.9)	
Lip notching	Yes	9 (11.4)	2 (5.7)	7 (15.9)	*p* = 0.012
	No	26 (32.9)	7 (20.0)	19 (43.2)	
Outcome (continuous variables)		**Mean ± stdev**	**Mean ± stdev**	**Mean ± stdev**	**P value (t-test)**
Length of stay (days)		21.7 ± 11.5	21.8 ± 11.7	21.6 ± 11.4	*p* = 0.921
Days to oral diet commencement		14.0 ± 8.6	15.2 ± 9.14	13.1 ± 8.19	*p* = 0.314
Returns to theatre (n)		0.6 ± 1.2	0.86 ± 1.46	0.41 ± 0.92	*p* = 0.100

## Discussion

In our retrospective study, 16% of head and neck reconstructive procedures included a lip split approach during the seven-year study period. A notable trend was observed in the evolution of surgical preference: from 2015 to 2018, predominantly lateral lip splits were performed (67.6%), while from 2019 to 2022 there were predominantly central lip splits (73.8%). This shift likely reflects surgeon adaptation to observed complication rates associated with lateral lip splits. Specifically, lateral lip splits were significantly associated with higher postoperative complications (*p* = 0.048), including double the rate of returns to theatre (50.0% vs. 25.6%; *p* = 0.025). Most returns to theatre were for recipient site complications (89% of returns to theatre) including collections such as hematomas, seromas, and abscess formation. Functionally, lip notching (*p* = 0.012) and asymmetric smiling (*p* = 0.009) were significantly more common in the lateral lip split cohort, while speech intelligibility (*p* = 0.522) and days to oral diet commencement (*p* = 0.417) were comparable between groups (*p* = 0.522).

Despite these findings, no significant differences were observed between the two cohorts in terms of readmission rates (*p* = 0.479) and mean length of stay (21.9 vs. 21.5 days; *p* = 0.870). These outcomes suggest that, while lateral lip splits are associated with a higher incidence of localised complications, they do not significantly influence the overall hospital course, likely due to the complexity of the patient cohort undergoing extensive surgery and reconstruction.

Lip splitting approaches to the oral cavity provide superior access for R_0_ tumour resection (i.e., microscopically clear margins)[4, 7, 12–14], with excellent visualization and handling of posterior structures [15, 16]. Recent literature has highlighted a decline in the use of lip-split techniques (with or without mandibulotomy) in favor of less invasive approaches [2, 11, 15]. Nevertheless, the lip-split remains an important tool for resection and reconstruction of many difficult to access oral and oropharyngeal tumors that may be inappropriate for alternative approaches due to anatomical or technical reasons [17].

The advent of free flap microsurgical reconstruction has further underscored the utility of lip split access. This technique enables precise deep intra-oral and pharyngeal flap inset and microsurgical vascular anastomosis [18]. A recent systematic review reported the outcomes of 3,872 patients (54 studies) that underwent lip-split and mandibulotomy access procedures [2]. The complication rates were low, with 5.4% osteoradionecrosis, 5.7% fistula formation, and 4.9% non-union. The authors concluded that lip-split mandibulotomy has an acceptable complication rate and should remain a viable surgical option, particularly when alternative lip-sparing approaches fail to provide sufficient exposure for tumour clearance [7].

To our knowledge there is only one other study comparing postoperative outcomes of midline and lateral lip-split. This small study evaluated patient satisfaction after central and lateral lip incisions, finding the chevron-chin modification of the McGregor midline approach as yielding optimal outcomes [19]. Lateral lip splits provide excellent access to many tumours of the oral cavity, including those located posteriorly (e.g. oropharynx) or laterally, but they frequently compromise neurovascular structures, including branches of the facial and mental nerves and the inferior and superior labial vessels [7]. This is particularly important if the contralateral labial vessel if small or absent, as the lip will be de-vascularised, leading to poor wound healing.

In contrast, central lip splits preserve the mental and marginal mandibular nerves, thereby reducing risk of paraesthesia. However, scarring following central lip incisions can lead to notching, fistula, and reduced lip mobility [7]. These risks are particularly pronounced in resections involving the anterior oral cavity and mandible, where residual central lip tissue may be devascularized during tumour resection. Consequently, the selection of a lip splitting approach should be based on a comprehensive assessment of tumour location, required resection margins, patient comorbidities, patient preferences, and surgeon preferences. For both techniques, meticulous closure of skin and mucosa with layers sutures is essential in optimizing functional and aesthetic outcomes.

Lip sparing approaches, including the visor technique and pull-through methods, have gained popularity as alternatives to lip-split procedures. While these techniques avoid lip incisions, they have their own limitations. The visor approach is undertaken with a mastoid-to-mastoid incision through a mid-neck crease. This approach leads to chin anaesthesia due to bilateral mental nerve sacrifice [10]. Two retrospective studies compared outcomes of lip splitting incisions with the lip sparing visor technique with no statistical difference in surgical-site complications nor operative duration (*p* > 0.05) [20, 21]. The visor approach can also be combined with lingual release for improved access. Focusing on aesthetic and functional outcomes, Devine et al. retrospectively compared 90 patients that underwent lip-split mandibulotomy with 60 patients undergoing a lingual-release [22]. Resection margins were similar, while the lip-split cohort had significantly better speech, swallowing, and chewing quality-of-life outcomes (*p* < 0.05). The poorer functional outcomes were thought to occur secondarily to the lingual release procedure in the visor incision cohort [22].

The pull-through technique involves detaching the anterior digastric and mylohyoid muscles and applying traction sutures to the tongue. Cheng et al. retrospectively compared lip-split surgery with a pull-through approach for T4a tongue and floor of mouth (FOM) cancer resection (*n* = 91)[23], noting difficulty with the pull-through technique in cases of limited mouth-opening or those requiring marginal mandibulectomy, and that flap inset was more technically difficult. Transoral laser surgery has also been compared with lip-split mandibulotomy in a small case-match study (*n* = 48) of oropharyngeal SCC resection with radial forearm free flap reconstruction [11].

There are several limitations to our study. Primarily the retrospective design limits direct comparison of groups. The small study size and significant patient complexity limited analysis of potential associations with smaller effect size. The significant shift from lateral to central lip-split approaches during the study period reflects evolving surgeon preferences, likely influenced by observed complications and anatomical considerations. Differences in tumour location, extent of resection, and reconstruction type further contribute to selection bias. Lateral lip splits were more frequently used in segmental mandibulectomies (*p* = 0.005) and inpatients with higher ASA classifications (*p* = 0.046), indicating a more complex cohort. Additionally, our inability to stratify outcomes by the type of reconstruction or by access-only compared to resection cases limits detailed analysis.

## Conclusion

Experienced surgeons may opt for transoral and transcervical approaches for resection and reconstruction of advanced oral and oropharyngeal carcinomas. However, the excellent access provided by lip splitting in these cases is unlikely to be superseded completely by these alternative approaches. Given its ongoing role in head and neck surgery, optimization of the lip split technique is warranted. This study of 79 patients undergoing lip-split access procedures for oral and oropharyngeal tumours demonstrates an association of lateral lip split access (e.g., the Robson approach) with poorer outcomes. Randomized prospective data is needed to compare approaches. This research should focus on surgical outcomes (including margin clearance, locoregional recurrence, and complications), functional outcomes (including speech and swallowing), and cosmetic outcomes including patient satisfaction.

## Data Availability

The data that support the findings of this study are available from the corresponding author upon reasonable request.
